# 

**Published:** 1888-09

**Authors:** 


### CONTENTS.

COMMUNICATIONS.

Anæsthetics in Dental Practice........................................ 421
Saliva........................................................................ 432
Artificial Vela for Congenital Cleft of the Palate.......................... 435
Tin and Gold Fillings.......................................................... 441

SELECTIONS.

A Contribution to the Study of Bone Repair.................................. 443
Cocaine in Surgery............................................................ 449
Pyorrhœa Alveolitis in the Elephant......................................... 454
Microscopy.................................................................... 455
Billings F. on three Recoveries from Tetanus................................ 456
Over-Population of the Towns................................................. 459
Lupus and Cutaneous Tuberculosis............................................ 460
The Time in which we Think................................................... 461
Delayed Dentition............................................................. 465
CORRESPONDENCE................................................................ 466

EDITORIAL.

Taggar's Corundum, Point and Disk maker...................................... 470
Obituary...................................................................... 472

